# Dietary Management and Nutritional Strategies for Preventing Age-Related Diseases

**DOI:** 10.3390/nu17091450

**Published:** 2025-04-25

**Authors:** Byungyong Ahn, Xian Wu, Yoo Kim

**Affiliations:** 1 Department of Food Science and Nutrition, University of Ulsan, Ulsan 44610, Republic of Korea; 2 Basic-Clinical Convergence Research Institute, University of Ulsan, Ulsan 44610, Republic of Korea; 3 Department of Kinesiology, Nutrition, and Health, Miami University, Oxford, OH 45056, USA; wux57@miamioh.edu; 4 Department of Nutritional Sciences, Oklahoma State University, Stillwater, OK 74078, USA

## 1. Introduction

The process of aging is a natural and inevitable biological occurrence that exerts a substantial influence on overall health and well-being [1]. As individuals advance in age, they become increasingly susceptible to a wide range of chronic diseases, including but not limited to cardiovascular diseases, neurodegenerative disorders such as Alzheimer’s and Parkinson’s disease, metabolic syndromes such as type 2 diabetes and obesity, musculoskeletal conditions, including osteoporosis and sarcopenia, and various types of cancer [2,3,4]. The increasing global life expectancy highlights the imperative for strategies that promote longevity and enhance quality of life. Among these strategies, effective dietary management stands at the forefront, offering a pivotal approach to mitigating the detrimental effects of aging while concurrently reducing the risk of developing chronic conditions [5]. Dietary and nutritional interventions are now widely recognized as effective means of influencing critical metabolic and cellular processes, encompassing inflammation, oxidative stress, mitochondrial function, and gut microbiota composition [6,7]. A nutritionally balanced diet meticulously tailored to meet the specific requirements of elderly individuals has been demonstrated to play a vital role in the prevention of frailty, cognitive decline, cardiovascular complications, and metabolic dysfunction, thereby promoting healthier living in later years [8,9].

In recent years, particular dietary components and patterns have garnered attention due to their capacity to enhance longevity and counteract age-related diseases. The Mediterranean diet, abundant in healthy fats, antioxidants, and anti-inflammatory compounds, has been extensively studied owing to its cardiovascular and cognitive benefits [10,11]. Controlled egg consumption has also been re-evaluated for its role in supplying vital nutrients while concurrently mitigating cardiovascular risks [12]. In addition, the dietary supplementation of creatine monohydrate has exhibited potential in maintaining muscle mass and cognitive function in older adults [13]. The exploration of bioactive compounds, including polyphenols, filbertone, curcumin, and essential vitamins, has been undertaken to assess their neuroprotective, anti-inflammatory, and metabolic regulatory properties [14,15,16,17]. Furthermore, the utilization of oral nutrition supplements has seen a marked increase in the context of addressing malnutrition and frailty in the elderly population [18].

The following Editorial examines recent discoveries in the field of nutritional science and their potential consequences for the management of age-related diseases. By means of a review of the latest studies on dietary interventions, including the Mediterranean diet, egg consumption, creatine monohydrate supplementation, filbertone, vitamin intake, curcumin, polyphenols, and oral nutrition supplements, the objective of the Editorial is to highlight practical and evidence-based dietary strategies that can contribute to healthier aging in older populations.

## 2. The Impact of the Mediterranean Diet, Vitamin Intake, and Egg Consumption on Frailty and Mortality in Older Adults

Frailty is a prevalent geriatric syndrome characterized by a decline in physiological resilience, leading to increased vulnerability to adverse health outcomes such as disability, hospitalization, and mortality. This condition is often associated with muscle weakness, fatigue, slowed mobility, and overall functional decline, which significantly impact the quality of life of older adults [19]. As the aging population continues to grow, researchers have focused on identifying dietary strategies that may mitigate frailty and promote healthier aging [20,21]. One promising dietary pattern in this regard is the Mediterranean diet, which has been extensively studied owing to its numerous health benefits, particularly in aging populations. In a recent study, Gross et al. examined the relationship between adherence to the Mediterranean diet and the risk of frailty using data from the National Health and Nutrition Examination Survey (NHANES) spanning from 2007 to 2017. Participants were categorized based on their level of adherence to the Mediterranean diet, and the findings revealed a significant inverse association between following this dietary pattern and the likelihood of developing frailty. Specifically, individuals with higher adherence to the Mediterranean diet were found to have substantially lower odds of becoming frail. These results highlight the crucial role of nutrition in maintaining muscle strength, reducing systemic inflammation, and supporting overall metabolic health in older adults [22]. Moreover, in another recent study, Park & Kim examined the relationship between vitamin intake and frailty in older adults who maintained adequate caloric and protein intake. Contrary to common assumptions, the study authors found no significant association between vitamin deficiencies and frailty in individuals with sufficient energy and protein intake. These findings suggest that while vitamins are essential for various metabolic and physiological processes, their role in preventing frailty may be less pronounced when an individual’s overall nutritional status is optimal. This finding underscores the importance of focusing on comprehensive dietary balance rather than relying solely on vitamin supplementation as a preventive strategy against frailty [23].

In addition, egg consumption has been a topic of ongoing debate in nutritional science due to its potential benefits and concerns related to cardiovascular health and longevity. Eggs are a nutrient-rich food, providing high-quality protein, essential vitamins, and bioactive compounds; however, concerns about their cholesterol content have led to conflicting dietary recommendations over the years. Understanding the role of egg consumption in aging populations is especially critical, as older adults require nutrient-dense foods to support muscle maintenance, cognitive function, and overall health. In a recent study, Wild et al. investigated the association between egg intake and mortality risk among older adults in Australia. The researchers analyzed data from a cohort of community-dwelling older individuals, examining the impact of different levels of egg consumption on all-cause and cardiovascular disease mortality. The findings revealed that moderate egg consumption—specifically between one and six times per week—was linked to a reduced risk of both all-cause mortality and cardiovascular mortality. These results suggest that eggs, when incorporated into a balanced diet, may contribute to improved health outcomes in older populations. Interestingly, the study authors also noted that daily egg consumption was associated with a slightly increased mortality risk. However, this association did not reach statistical significance, indicating that more research is required to determine whether frequent egg consumption poses long-term health risks [24].

## 3. Creatine Supplementation and Oral Nutrition: Enhancing Vascular Function and Physical Performance in Older Adults

Creatine monohydrate is widely recognized for its benefits in enhancing muscle strength, power output, and overall physical performance. While traditionally used as a supplement among athletes and individuals engaged in resistance training, creatine has gained increasing attention for its potential therapeutic applications in aging populations. Although its role in muscle health is well documented, emerging research suggests that creatine monohydrate supplementation may also have positive effects on vascular function and metabolic health in older adults [25]. In a recent pilot study conducted by Clarke et al., the authors investigated the impact of creatine monohydrate supplementation on endothelial function in aging individuals. Endothelial function plays a critical role in vascular health, influencing blood flow regulation, inflammation, and cardiovascular disease risk. The study authors evaluated key markers such as vascular function, including macrovascular function, microvascular reperfusion rates, and metabolic parameters, including fasting glucose and triglyceride levels. After four weeks of supplementation, participants demonstrated significant improvements in macrovascular function and enhanced microvascular reperfusion rates, suggesting better circulation and oxygen delivery to tissues. Reductions in fasting glucose and triglyceride levels also suggested potential metabolic benefits, which could be particularly relevant for older adults at risk of insulin resistance and cardiovascular disease [26].

In addition, malnutrition is a widespread issue among older adults, particularly those residing in nursing homes or long-term care facilities. Poor dietary intake can lead to muscle loss, decreased functional capacity, and increased risk of frailty and morbidity [27]. Addressing nutritional deficiencies through targeted interventions is crucial in enhancing the quality of life and physical performance of aging individuals. In a recent study, Chen et al. (2024) investigated the effects of oral nutrition supplements (ONSs) on nutritional status and physical performance in nursing home residents. The findings revealed that ONS intervention led to significant improvements in body weight, walking speed, and overall quality of life among participants. These results underscore the importance of providing tailored nutritional support to older adults, particularly those at risk of malnutrition. Oral nutrition supplements offer a practical solution for individuals who struggle to meet their dietary needs through regular food intake alone [28]. Incorporating ONSs into elderly care strategies can help prevent muscle wasting, enhance mobility, and reduce the risk of hospitalization due to malnutrition-related complications. However, personalized dietary plans should be developed to ensure that ONSs are used effectively, considering factors such as individual energy requirements, underlying health conditions, and dietary preferences.

## 4. Natural Compounds in Aging: Filbertone for Muscle Health, Curcumin for Cognitive Function, and Polyphenols for Gut Microbiota

Muscle aging is a major health concern for older adults, as it contributes to reduced mobility, increased frailty, and heightened risk of falls and disability. Sarcopenia, defined as the age-related loss of muscle mass and strength, affects millions of people worldwide and has profound implications for overall health and quality of life [29]. The results of recent studies highlight filbertone, a bioactive compound in hazelnuts, as a potential solution for preserving muscle health. In a recent study, Jung & Ahn investigated the effects of filbertone on aging skeletal muscle cells. The researchers found that filbertone supplementation reduced markers of cellular senescence, a key factor in muscle aging, while concurrently enhancing the expression of muscle-related genes. These findings suggest that filbertone may help maintain muscle integrity, delay deterioration, and support overall musculoskeletal health [30]. While these initial findings are promising, further research is needed to confirm the efficacy of filbertone in humans. In future clinical trials, its effects on muscle mass, strength, and functional performance should be investigated in aging individuals. In addition, researchers should perform studies to determine optimal dietary sources and dosages to maximize its benefits. As filbertone is naturally found in hazelnuts, the inclusion of hazelnuts or their extracts in the diet may be a practical, nutrition-based approach to muscle preservation in older adults.

As our populations grow older, cognitive decline and neurodegenerative disorders, e.g., Alzheimer’s disease, pose significant challenges. Accordingly, there is growing interest in potential dietary interventions to support brain health. Curcumin has been identified as a promising agent due to its multifaceted properties, which include strong anti-inflammatory, antioxidant, and gut-modulating effects [31]. In a recent investigation, Lamichhane et al. examined the impact of curcumin on cognitive function and metabolic health in a mouse model of Alzheimer’s disease induced by a diet high in fat and sugar. The findings of this investigation revealed that curcumin supplementation can ameliorate impairments in spatial memory, enhance hepatic metabolism, and exert a favorable influence on gut microbiome composition. They also suggest the possibility of a protective role for curcumin in Alzheimer’s disease via the modulation of key pathways involved in synaptic plasticity, neuroinflammation, and gut–brain axis interactions [32].

The gut microbiota has been identified as a significant factor in overall health, impacting metabolic processes, immune function, and even cognitive health. As individuals age, the composition of the gut microbiota changes significantly, often leading to dysbiosis, a microbial imbalance associated with increased inflammation, metabolic disorders, and neurodegenerative diseases [33]. Given the mounting evidence supporting the concept of a bidirectional relationship between the gastrointestinal tract and the brain in the process of aging and the development of cognitive impairment, the implementation of dietary strategies that are designed to modify the gut microbiota has become a subject of considerable research interest. In a recent review, Pereira and colleagues explored the role of polyphenolic compounds in maintaining gut microbiota balance and promoting healthy aging. Polyphenols, which are present in a variety of plant-based foods such as berries, green tea, cocoa, and red wine, have been shown to possess strong antioxidant and anti-inflammatory properties. In their review, the authors emphasized that polyphenols can exert a favorable influence on gut microbiome composition by promoting the growth of beneficial bacteria while impeding the proliferation of potentially harmful species. These effects may have far-reaching implications for aging-related health outcomes, including reduced systemic inflammation, improved metabolic function, and enhanced cognitive resilience. Modulation of the gut microbiota by polyphenols has the potential to contribute to the prevention of age-related diseases, including type 2 diabetes, cardiovascular diseases, neurodegenerative disorders, and cancer [34].

As global life expectancy continues to rise, the necessity for efficacious nutritional strategies to support healthy aging is becoming ever more apparent. Scientific research findings have demonstrated the crucial role of dietary interventions in the mitigation of age-related diseases and the improvement of overall quality of life. A substantial body of evidence has emerged to support the notion that a Mediterranean diet can confer a degree of protection against the development of frailty, while the ingestion of creatine monohydrate has shown promise in enhancing vascular function. These observations underscore the critical importance of evidence-based dietary guidelines tailored to meet the specific nutritional needs of the aging population. A diet that includes moderate consumption of eggs has been associated with a reduced mortality risk. Bioactive compounds, such as filbertone and curcumin, show promise in supporting muscle and cognitive health. Furthermore, oral nutritional supplements have the capacity to deliver targeted nutritional support to older adults, while a dietary approach involving the consumption of polyphenols can assist in maintaining a healthy gut microbiota balance and preventing age-related metabolic and neurodegenerative disorders. Despite these findings providing valuable insights into the relationship between diet and ageing, further longitudinal and interventional studies are necessary to refine evidence-based dietary guidelines tailored to older populations. The multifaceted nature of age-related diseases necessitates a personalized approach, encompassing diverse nutritional regimens, bioactive compounds, and supplementation methodologies to address the distinct requirements of each individual.
